# Integration of Zeolite Membrane Bioreactor With Granular Sludge-Based Anammox in High-Efficiency Nitrogen Removal From Iron Oxide Red Wastewater

**DOI:** 10.3389/fmicb.2022.932940

**Published:** 2022-06-29

**Authors:** Xing-Hui Feng, Xiao-Jun Wang, Hai-Xiang Li, Hai-Ya Zhang, Zong-Qiang Zhu, Yan-Peng Liang, Kun Dong, Hong-Hu Zeng

**Affiliations:** ^1^School of Environmental Science and Engineering, Guilin University of Technology, Guilin, China; ^2^Collaborative Innovation Center for Water Pollution Control and Water Safety in Karst Area, Guilin University of Technology, Guilin, China; ^3^School of Environment and Energy, South China University of Technology, Guangzhou, China; ^4^Institute of Water Ecology and Environment, Chinese Research Academy of Environmental Science, Beijing, China

**Keywords:** nitrogen removal, PN-anammox, granular sludge, IORW, alkalinity

## Abstract

Acquisition of stable nitritation and efficient anammox play a crucial role in partial nitritation (PN) combined with anammox for nitrogen removal from ammonium-rich wastewater. Due to the limitation of ammonia-oxidizing bacteria (AOB) enrichment and nitrite-oxidizing bacteria (NOB) control in traditional membrane biological reactor (MBR), it can result in a lower nitrite production rate (NPR) and unstable PN, eventually reducing the nitrogen removal rate (NRR) *via* PN-anammox. In this study, we developed a zeolite membrane biological reactor (ZMBR) to enhance the PN of iron oxide red wastewater (IORW), in which the biofilm derived from the zeolite surface can provide free ammonia (FA)-containing microenvironment for AOB enrichment and NOB inhibition. The results showed that ZMBR can tolerate a higher influent nitrogen loading rate (NLR) of 2.78 kg/(m^3^⋅day) in comparison to the traditional MBR [2.02 kg/(m^3^⋅day)] and the NPR in ZMBR and traditional MBR were 1.39 and 0.96 kg/(m^3^⋅day), respectively. The mass concentration ratio of ${\text{NO}}_{2}^{-}$-N/${\text{NH}}_{4}^{+}$-N ranged from 1.05 to 1.33 in ZMBR, suggesting a suitable condition for nitrogen removal *via* anammox. Subsequently, the domesticated granular sludge obtained from a paper-making wastewater treatment was used as the carrier of anammox bacteria to remove nitrogen. After 93 days of operation, the NRR was observed to be 2.33 kg/(m^3^⋅day) and high-throughput sequencing indicated that the relatively higher abundance (45.0%) of *Candidatus* Kuenenia stuttgartiensis was detected in the granular sludge of the bottom part of the reactor, which can produce more proteins and lipids, suggesting a good settleability. Overall, this study provides a high-efficient method to control PN and domesticate anammox for nitrogen removal from IORW.

## Introduction

About 15 tons of iron oxide red wastewater (IORW) occurred per ton of finished product, which contains lots of ammonium (${\text{NH}}_{4}^{+}$-N), Fe^2+^, and ${\text{SO}}_{4}^{2-}$. The previous study has proved that aeration oxidation and alkaline neutralization are effective solutions for removing Fe^2+^ from which iron mud can be recycled and used as the inoculating crystal (Feng et al., 2019). However, the remaining ${\text{NH}}_{4}^{+}$-N is at high concentrations (700–1,000 mg/L) in the supernatant. The improper management or direct discharge of IORW could cause serious water eutrophication; accordingly, a low-carbon and low-cost technology for nitrogen removal is a significant requirement. Due to the presence of ${\text{SO}}_{4}^{2-}$, the traditional nitrification-denitrification technology might not be an acceptable process for nitrogen removal because sulfate-reducing bacteria (SRB) convert sulfate to H_2_S, which would lead to serious secondary pollution and inhibition of nitrification.

Recently, the partial nitritation (PN) combined with anaerobic ammonium oxidation (PN-anammox) has been widely used in nitrogen removal system *via* anammox by using ${\text{NH}}_{4}^{+}$-N as the electron donor and ${\text{NO}}_{2}^{-}$-N as the electron acceptor, and its engineering application, is drawing increasing attention from researchers. The PN-anammox reduces the need for organic carbon and aeration and decreases sludge production compared with traditional nitrification-denitrification (Lackner et al., 2014). Currently, a limiting step in PN-anammox is to obtain stable PN. Generally, ammonia-oxidizing bacteria (AOB) and nitrite-oxidizing bacteria (NOB) play a crucial role in the nitritation and nitratation process, respectively (Sinha and Annachhatre, 2007; Laureni et al., 2016). Nitrite accumulation depends on nitritation (Anthonisen et al., 1976) and AOB abundance increased, while NOB number decreased at high ammonium loading conditions (Shao et al., 2018). The literature reports numerous indirect methods to maintain effective AOB and inhibit NOB in the same reactor, for instance, controlling free nitrous acid (FNA) (Pedrouso et al., 2017), maintaining a low concentration of dissolved oxygen (DO) in a sequencing batch reactor (SBR) (Li et al., 2011) and regulating free ammonia (FA) in zeolite biological aeration filter (ZBAF). A previous study investigated the PN in two-stage ZBAF by controlling the alkalinity dosage resulting that stable PN being obtained in this combined system (Feng et al., 2019). In addition, high FA induced by Na_2_CO_3_ was avoided resulting that Na_2_CO_3_ as an alkalinity donor could save about 40% dosage than common NaHCO_3_. Although ZBAF can be stable and low-cost in the PN from IORW, the low treatment efficiency and frequent backwashing requirement result that it might not be suitable for large-scale implementation. Moreover, the detached biofilm from zeolite would outflow along with the affluent, affecting the substance concentration (nitrite and ammonium). Besides, numerous theoretical and practical applications claim that membrane biological reactors (MBRs) have advantages in higher volumetric loading, better effluent quality, and less sludge loss (Barreto et al., 2017). These advantages from MBRs coupled with zeolite to maintain high-efficiency PN and used Na_2_CO_3_ as the alkalinity donor, which should have a wide market foreground and important engineering significance for IORW treatment. However, the premise is to achieve stable PN in a zeolite membrane biological reactor (ZMBR).

Anammox bacteria cultivated by feeding with real wastewater is another key step in nitrogen removal from IORW treatment because it has a high sensitivity to changing environmental conditions, which makes their cultivation extremely difficult (Ni and Meng, 2011). Besides, the growth rate of anammox bacteria is regarded as restrictive to engineering applications (Jin et al., 2012). Nitrogen removal efficiency is the common parameter to reflect anammox activity. The literature reports the different doubling times of anammox bacteria as 10–14 (Marc et al., 2006), 4.8 (Tang et al., 2011), and 1.8 days (Isaka et al., 2006). Different sludge morphologies (e.g., floc and granule) have different adhesive surfaces, which have differences in anammox bacteria enrichment. The characteristics of granular sludge mainly include well-defined shape, high settleability, enhanced microbial activities, and its application in domestic or industrial wastewater treatment (Sarma et al., 2017). Although flocculent sludge has a larger specific surface area that could offer more attachment sites for anammox bacteria than granular sludge, sludge loss would be a challenge in practical application. Moreover, extracellular polymeric substances (EPSs) are usually found in the metabolic activities of microorganisms and look like a gel-like network on the surface of granules and play a crucial role in maintaining their structural stability (Cai et al., 2017). Especially the proteins, lipids, and β-D-mannose are also beneficial to maintaining sludge structure and promoting sludge aggregation (Jia et al., 2017). In other words, EPS might be beneficial for the attachment of microorganisms to the sludge. Granular sludge is often considered a typical solid waste that needs reasonable disposal in papermaking plants. Because of this, using granular sludge as the carrier of anammox bacteria is also a potential resource utilization method, which still needs experimental verification.

In this study, the PN of IORW was conducted in ZMBR, followed by using granular sludge-based anammox for further nitrogen removal. The objective of this study was to: (1) obtain controllable PN of IORW in ZMBR and evaluate the biofilm formation on the surface of zeolite; (2) recycle granular sludge from the papermaking wastewater treatment process for the carrier of anammox bacteria; (3) investigate the living environment of anammox bacteria through EPS (proteins, β-D-mannose, and lipid) detection by using confocal laser scanning microscopy (CLSM); and (4) use ZMBR combined with granular sludge-based anammox for the nitrogen removal from IORW. The obtained result might provide a high-efficiency and cost-effective strategy for IORW treatment.

## Materials and Methods

### Experimental Design

**FIGURE 1:**
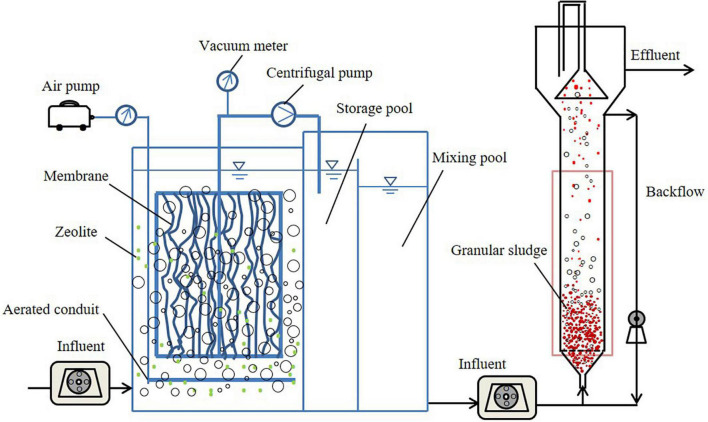
Schematic diagram of the partial nitritation (PN)-anammox process.

The ZMBR was designed as a rectangular vessel made of stalinite with a total volume of 10 L (working volume was 8 L) and the surface area of microfiltration polymeric membranes was 2 m^2^ (the ratio of the addition of membrane to the working volume of the reactor was 0.25 m^2^/L). Natural zeolite was filled as the ${\text{NH}}_{4}^{+}$-N adsorbents that the average particle size was 0.5–1 mm (diameter) and the filling rate was ranged from 2 to 5% of the reactor volume. Based on upflow anaerobic sludge blanket (UASB) and anammox reactors, they were made of transparent acrylic plates of 1,000 mm in height and 80 mm in diameter. The total volume was 5 L and the sludge volume was about 1.5 L. The schematic diagram of the PN-anammox process is shown in Figure 1.

### Experimental Wastewater and Sludge

Test wastewater was taken from the pretreatment process of an iron oxide red plant (Jiangmen, China) which has completed Fe^2+^ removal under the condition of aeration and added NaOH then static settlement. The concentration of ${\text{NH}}_{4}^{+}$-N was between 700 and 1,000 mg/L and ${\text{SO}}_{4}^{2-}$ was in the range of 8,000–10,000 mg/L in the supernatant.

The seed sludge of nitritation was obtained from a refuse landfill (Guangzhou, China) and using clean water washed several times then cultivated under the laboratory condition (feed with alkalinity and ${\text{NH}}_{4}^{+}$-N). The granular sludge came from the sewage treatment process of a papermaking plant (Liuzhou, China). The seed sludge of anammox was taken from an anammox reactor that was fed with synthetic water in a laboratory and the dominant anammox bacteria were *Candidatus* Brocadia sinica and *Candidatus* Kuenenia stuttgartiensis.

### Operational Strategies

The ZMBR startup was divided into three phases: Phase I, ${\text{NH}}_{4}^{+}$-N was enriched in the zeolite *via* adsorption; phase II, added 2 L seed sludge to activate PN [the mixed liquid suspended solids (MLSSs) was about 3,000 mg/L]; and phase III, gradually increase the influent ${\text{NH}}_{4}^{+}$-N concentration or shortening the hydraulic retention time (HRT) to increase influent nitrogen loading rate (NLR). Using an air pump to maintain the DO concentration in the range of 4–6 mg/L and the operating temperature was 28 ± 2°C in the ZMBR.

Using MgSO_4_ (0.1 mg/L), KH_2_PO_4_ (0.05 mg/L) to provide essential microelement and Na_2_CO_3_ as alkalinity donor. According to the previous study, alkalinity/${\text{NH}}_{4}^{+}$-N = 4.33 (mass concentration ratio) was an optimum ratio to obtain ${\text{NO}}_{2}^{-}$-N/${\text{NH}}_{4}^{+}$-N = 1.0–1.4 that is suitable for further treatment *via* anammox (Feng et al., 2019). The concentrations of ${\text{NH}}_{4}^{+}$-N, ${\text{NO}}_{2}^{-}$-N, and ${\text{NO}}_{3}^{-}$-N were detected in every 12 h to assess the nitritation efficiency and FA (Anthonisen et al., 1976), nitrite production rate (NPR), NLR, and nitrogen removal rate (NRR) were calculated by the following equations:


(1) $$\text{FA}\,\left(m\,g/L\right)=\frac{17}{14}\times \frac{N\,H_{4}^{+}-N\times 10^{p\,H}}{\exp\left[6334/\left(273+T\right)\right]+10^{p\,H}}$$

(2) $$\text{NPR}\,\left[\mathrm{kg}\,N/\left(m^{3}\cdot \text{d}\right)\right]=24\times \frac{N\,O_{2}^{-}-N_{e\,f\,f}}{H\,R\,T}$$

(3) $$\text{NLR}\,\left[\mathrm{kg}\,N/\left(m^{3}\cdot \text{d}\right)\right]=24\times \frac{T\,N_{i\,n}}{H\,R\,T}$$

(4) $$\text{NRR}\,\left[\mathrm{kg}\,N/\left(m^{3}\cdot \text{d}\right)\right]=24\times \frac{T\,N_{i\,n}-T\,N_{e\,f\,f}}{H\,R\,T}$$

Anammox reactor was started up by inoculating with anammox sludge, increasing the influent substrate concentration (${\text{NH}}_{4}^{+}$-N and ${\text{NO}}_{2}^{-}$-N), and put on a heating jacket to maintain the temperature between 28 and 32°C.

### Quantitative PCR Assays

Absolute quantification-PCR (AQ-PCR) technology was based on the known copy number variation (CNV) from the standard sample to obtain a standard curve. According to the cycle threshold (Ct) value, the absolute quantification of CNV from messenger RNA (mRNA) or DNA could be calculated according to the standard curve (Walker, 2001). Because of the relatively low abundance in NOB, it results that it was usually not detected by the common high-throughput sequencing technology. Therefore, PCR was employed to detect the relative ratio of AOB and NOB, which coexistence on the biofilm of zeolite.


$$\text{Ct}=-\text{Klog}\,X_{0}+\text{b}$$

*Ct* refers to the cycle index when the fluorescence signal of the amplified product reaches the set threshold value and *K* was the slope and *b* as the y-intercept of standard curve.

This detection was supported by the Shanghai Sunny Biotechnology Corporation Ltd. The related primer information of AOB and NOB is given in Table 1. The real-time PCR samples were carried out by using 20 μl reaction volumes that contained 10 μl mixture A, which consist of 10 μl 2 × SYBR real-time PCR premixture and 0.4 μl from primer-F and -R (10 μM, the primer information from AOB and NOB was found in Table 1), 10 μl DNA diluent as template (using DNA extraction kit obtained 1.61 μg DNA in 50 μl solution, cat: DP305, TianGen Biotechnology Corporation Ltd., Beijing, China). The thermal cycling was performed under the following conditions: 95°C for 5 min, followed by 40 cycles of 95°C for 15 s, and a final extension at 60°C for 30 s. All the operations, primers, and analysis results were provided by Sunny Biotechnology Corporation Ltd. (Shanghai, China).

**TABLE 1:** The related primer information of ammonia-oxidizing bacteria (AOB) and nitrite-oxidizing bacteria (NOB).

ID	Primer	Sequence(5′ to 3′)
amoA (AOB)	amoA-F	GGGGTTTCTACTGGTGGT
	amoA-R	CCCCTCKGSAAAGCCTTCTTC
*Nitrobacter* sp. 16S (NOB)	NxrB-F	ACGTGGAGACCAAGCCGGC
	NxrB-R	CCGTGCTGTTGAYCTCGTTGA
*Nitrospira* sp. 16S (NOB)	NSR-F	CCTGCTTTCAGTTGCTACCG
	NSR-R	GTTTGCAGCGCTTTGTACCG

### Confocal Laser Scanning Microscopy Observation

The samples from the anammox reactor were pretreated by staining and then observed by CLSM to detect the EPS (proteins, β-D-mannose, and lipid). The procedures of staining were adopted from a reference (Wu et al., 2018) and with a little modification that is described as follows: (1) Fluorescein isothiocyanate (FITC) (CAS No. 27072-45-3, Macklin, Shanghai, China) was applied to stain proteins; (2) Calcofluor white (CalW) (CAS No. 4404-43-47, Sigma-Aldrich Corporation, California, CA, United States) for β-D-mannose detection; and (3) Nile Red (CAS No. 7385-67-3, Macklin, Shanghai, China) was used to stain lipids.

The detailed staining steps were followed by adding a drop of FITC (10 mg/L, dimethyl sulfoxide as solvent) to the samples standing for 30 min and washing for three times by using phosphate-buffered saline (PBS) (0.01 M, pH = 7.4). Then, using CalW solution combined with 10% KOH (1:1) stained 1 min and washed with PBS several times. Next, put the samples in Nile red solution (5 mg/L, methanol as solvent) to stain for 5 min. Finally, a drop of the antifade mounting medium was added to the test samples. After staining, using PBS washing then put on the glass slide and observed by CLSM (Leica TCS SP8, Germany) and amplified about 4–10 times. The measuring parameter and observable color from different staining conditions are given in Table 2.

**TABLE 2:** Operation parameter and observable color of detection.

Dye	Emission wavelength (nm)	Excitation wavelength (nm)	Color
FITC	488	500–550	Green
CalW	400	410–480	Blue
Nile red	514	625–700	Red

### Chemical Analyses

Based on the standard examination of water and wastewater (Walter, 1961), the concentrations of ammonium, nitrite, and nitrate were periodically detected to evaluate the nitrogen removal performance and an automatic potentiometric titration was applied for the alkalinity measurement (based on CaCO_3_ calculation, mg/L). A multifunctional portable instrument (HQ30d, HACH) was employed for the detection of temperature and DO. A pH meter (PHS-3C) was used to obtain the pH value. Using high-throughput sequencing technology, it investigates the bacterial community structure in sludge samples [this part was supported by The Beijing Genomics Institute (BGI) Corporation Ltd.].

## Results

### Partial Nitritation Assessments in Zeolite Membrane Biological Reactor

**FIGURE 2:**
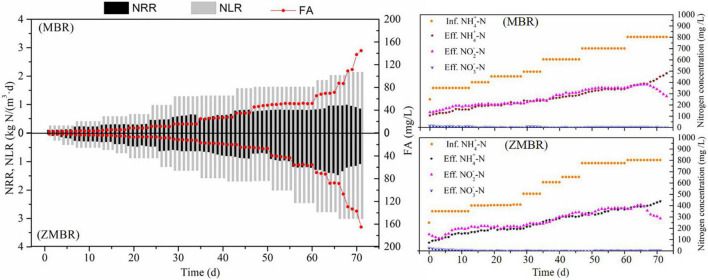
Evaluation of the PN, free ammonia (FA), and the nitrogen concentrations in MBR and zeolite membrane biological reactor (ZMBR).

Partial nitritation was maintained in ZMBR by gradually increasing the influent ammonium concentration and shortening HRT. As shown in Figure 2, ZMBR exhibited excellent nitritation efficiency and obtained 2.78 kg/(m^3^⋅day) in NLR and 1.40 kg/(m^3^⋅day) in NPR by influent ${\text{NH}}_{4}^{+}$-N of 803 mg/L, while the MBR without zeolite achieved 2.02 kg/(m^3^⋅day) (NLR) and NPR was 0.96 kg/(m^3^⋅day). As compared to a previous study, it showed that the maximum influent NLR from one-stage ZBAF was 1.20 kg/(m^3^⋅day) and obtained 0.76 kg/(m^3^⋅day) in NPR by influent 550 mg/L ${\text{NH}}_{4}^{+}$-N from synthetic wastewater (Feng et al., 2019). The PN performed in two-stage ZBAF achieved the NPR up to 0.67 kg/(m^3^⋅day) by influent 1.46 kg/(m^3^⋅day) NLR at 752 mg/L ${\text{NH}}_{4}^{+}$-N from IORW (Feng et al., 2019). Dramatically, the PN in non-zeolite MBR would be seriously inhibited, if NLR was up to 2.14 kg/(m^3^⋅day) then nitritation was gradually inhibited and FA increase to 109.0 mg/L. However, it would not be inhibited until the NLR is high to 3.01 kg/(m^3^⋅day) in ZMBR and the corresponding FA was 88.5 mg/L. Due to the higher NLR maintained in ZMR, more DO was consumed to support high-efficient nitritation, which resulted that DO concentration in ZMBR (4–6 mg/L) being higher than ZBAF (3–6 mg/L). Moreover, PN in ZMBR can obtain the range of ${\text{NO}}_{2}^{-}$-N/${\text{NH}}_{4}^{+}$-N ratio was 1.07–1.33, which would provide the necessary influent substrate ratio for nitrogen removal *via* anammox. Stable PN is maintained in ZMBR, which can support nitrogen removal from high ammonium wastewater and profit by the low carbon and energy-saving of anammox.

In the PN phase, zeolite and biofilm played the role of ${\text{NH}}_{4}^{+}$-N adsorption-desorption and N biochemical conversion, respectively, which can be a key factor in maintaining high-efficiency PN. Due to zeolite, a special adsorptivity to ammonium and the FA concentration in liquid were lower than that on the surface of zeolite; therefore, AOB would easily grow on the biofilm than in suspended sludge. The biofilm on the surface of zeolite formed by different microorganisms and the dominant bacterial community (AOB) might be valid evidence to hold the efficient nitritation. According to the amplification and copy numbers in the biofilm from the surface of zeolite (Table 3), NOB can coexist with AOB on the biofilm and more copy numbers were obtained from AOB than from NOB. Since the FA inhibited NOB and AOB kept working, the ZMBR profited from the special existence of zeolite that supported a higher influent loading than the MBR.

**TABLE 3:** Copy numbers of AOB and NOB (calculated by standard curve).

Gene	Ct	Standard curve	*R*	X_0_(copy/g)	SD
amoA	13.56	Ct = −3.19log*X_0_* + 34.83	0.986	2.40 × 10^9^	1.42 × 10^7^
*Nitrobacter* sp. 16S	19.9	Ct = −3.21log*X_0_* + 35.74	0.983	7.75 × 10^7^	3.64 × 10^6^
*Nitrospira* sp. 16S	29.20	Ct = −3.18log*X_0_* + 33.10	0.996	1.30 × 10^4^	0.85 × 10^3^

### Appraisal of the Granular Sludge-Based Anammox

**FIGURE 3:**
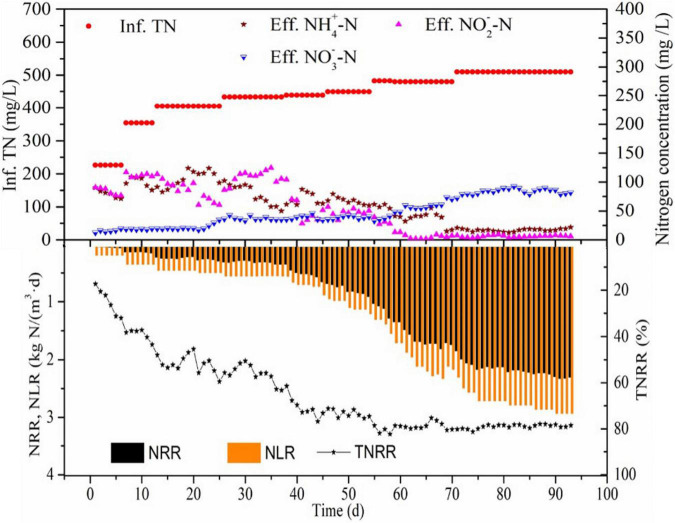
Nitrogen removal from granular sludge-based anammox reactor.

Anammox bacteria are belonging to branching from Planctomycetes (Kuenen, 2008). At present, *Candidatus* Brocadia sinica, *Ca.* K. stuttgartiensis, *Candidatus* Jettenia asiatica, *Candidatus* Scalindua wagneri, and *Candidatus* Anammoxoglobus propionicus had been identified as the effective bacteria in anammox process (Hu et al., 2011; Mamoru et al., 2011). Microbial analysis at different taxonomy levels provides a further understanding of bacterial community function. *Ca*. K. stuttgartiensis can rapidly enrich in the IORW treatment process and dominate in granular sludge. The literature reports that *Ca*. K. stuttgartiensis has advantages in adapting to the environment of the nitrite-limiting environment (Park et al., 2015), while the other four prefer to grow in the substrate-rich anammox-based process (Gonzalezgil et al., 2015). Besides, sludge structure, inoculum, and organic matter might be other potential factors that affect bacterial community structure (Date et al., 2009). IORW is typical industrial wastewater and the possible affecting factors to anammox still need deep analysis. Generally, anammox was started by using seed sludge to shorten the startup time and rapidly increased treatment efficiency. In this study, granular sludge was obtained from a papermaking wastewater treatment process, which was applied as the microbial carrier and seeded with anammox sludge to further remove ammonium and nitrite. In the first 47 days, influent TN concentration was 226–450 mg/L (the ${\text{NO}}_{2}^{-}$-N/${\text{NH}}_{4}^{+}$-N ratio was in the range of 1.07–1.33) and effective nitrogen removal was achieved. As shown in Figure 3, the granular sludge-based anammox achieved stable nitrogen removal efficiency after 93 days’ operation by influent IORW and the corresponding NRR was 2.33 kg/(m^3^⋅day). The literature reports that overloads lead to system instability and the anammox process would be seriously inhibited when the nitrite concentration is higher than 280 mg/L (Trigo et al., 2006; Isaka et al., 2007).

### Evolution of Microbial Community on Naturalized Granular Sludge

**FIGURE 4:**
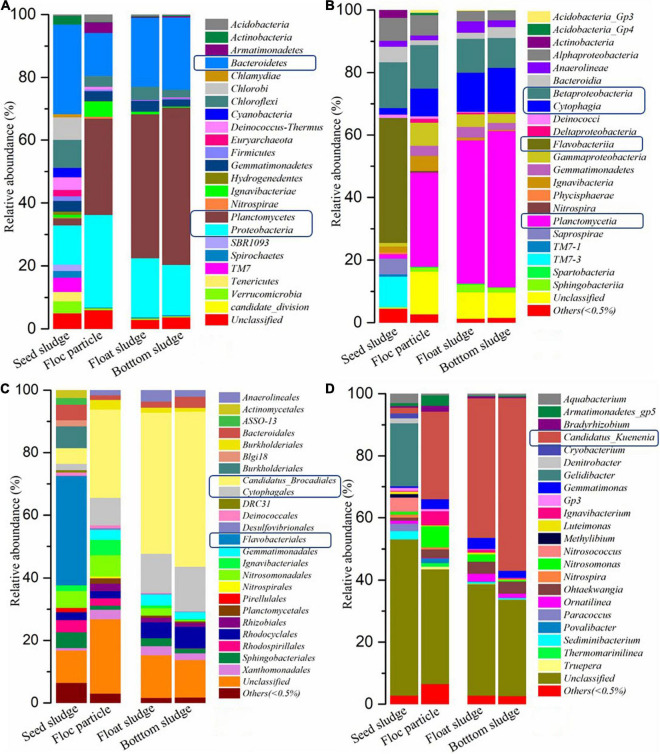
Microbial community structure and relative abundance in different sludge samples. Taxonomy in phylum **(A)**, class **(B)**, order **(C)**, and genus **(D)**.

The granular sludge was used as a carrier of anammox bacteria in the further nitrogen removal process, which was obtained from a papermaking wastewater treatment process. According to the high-throughput sequencing detection, 632 operational taxonomic units (OTUs) and 1,093 OTUs were obtained from seed sludge samples and cultivating sludge in 90-day samples, respectively. Based on the classification at different taxonomy levels, the bacterial community structure from floc particle (settle at the bottom of the reactor), float sludge (floating on the surface of the liquid), and bottom sludge (accumulate at the bottom of the reactor) were similar, while the relative abundance exhibited significant difference when achieved stable anammox. As shown in Figure 4, the four most abundant bacteria at the phylum level were *Bacteroidetes* (28.5%), *Proteobacteria* (12.6%), *Chloroflexi* (8.9%), and *Chlorobi* (7.2%) in seed sludge sample, while it changed to *Planctomycetes*, *Proteobacteria*, and *Bacteroidetes* in the samples from floc particle, float sludge, and bottom sludge after 90 days of operation. Compared to the seed sludge, an obvious increase can be seen in *Planctomycetes* and *Proteobacteria*, which proved that anammox bacteria gradually increase on the granular sludge.

The granular sludge from the bottom has the most abundant *Ca.* K. stuttgartiensis (56.6%), followed by fine particle granular sludge (45.6%) and the last was float sludge (45.1%). The influent substances were from the bottom to the top (UASB) and the bottom sludge preferential using the substances than the floating sludge, which might be the reason for the different content of anammox bacteria. The reference reported that *Ca.* B. sinica and *Ca.* K. stuttgartiensis are usually dominant in large granules (greater than 400 μm in diameter) and *Ca.* J. asiatica was enriched in fine particles (less than 200 μm in diameter) that were cultivated with synthetic water (Liu et al., 2017). However, this study showed obvious differences compared to the reference because of different wastewater treatment and inoculation. In addition, the possible hydraulic and pneumatic shock cause channeling phenomenon with the result that substances maldistribution. This might easily lead to the different microenvironments with a high concentration of substances or severe shortage of substances concurrence. The other literature reports that the anammox activity would be completely lost when the anammox biomass aggregates exposure to a ${\text{NO}}_{2}^{-}$-N solution of 100 mg/L (Van De Graaf et al., 1995) and the other results show that the ${\text{NO}}_{2}^{-}$-N concentration of 40 mg/L over several days would lead to an irreversible inactivation (Hulle et al., 2010). This study believed that the tolerance to ${\text{NO}}_{2}^{-}$-N concentration depends on the relative abundance of anammox bacteria. Moreover, substance distribution was another important factor in anammox bacteria enrichment.

## Discussion

### Zeolite Plays a Key Role to Build a Microenvironment of High Free Ammonia

**FIGURE 5:**
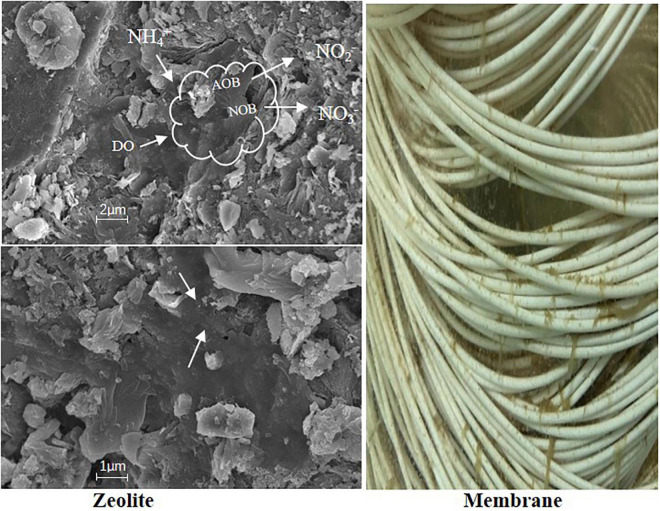
Biofilm formation on the surface of zeolite and membrane.

Zeolite has a special characteristic of absorption-desorption to ${\text{NH}}_{4}^{+}$-N, which should be the reason for the better performance than the process without zeolite. Besides, this kind of particularity can avoid ${\text{NH}}_{4}^{+}$-N substantial increase or decrease. Therefore, the ZMBR exhibited higher nitritation efficiency than the MBR by the same influent NLR, which might benefit from the stable concentration of ${\text{NH}}_{4}^{+}$-N and FA. As shown in Figure 5, zeolite was an efficient carrier that could maintain large areas of biofilm and lots of microorganisms attached to the biofilm and the biofilm formation on the zeolite might reduce a large amount of biofilm attached to the membranes. Moreover, the friction effect of the interfacial concrete from zeolite and membrane under aeration conditions might be another factor affecting a large amount of biofilm formation on the membrane. According to the above, the ZMBR obtained higher treatment efficiency than the MBR in the PN of IORW. Compared to the PN in a one-stage or two-stage ZBAF and SBR, the ZMBR has higher treatment efficiency and effluent quality. In addition, a previous study reported that using Na_2_CO_3_ as the alkalinity donor for PN could save about 40% dosage than common NaHCO_3_, while it would bring higher FA and cause inhibition to PN (Feng et al., 2019). Thus, cost efficiency and high efficiency were achieved in the practical application. In this study, the ZMBR could maintain higher efficiency for PN than the MBR or ZBAF by using Na_2_CO_3_ as the alkalinity donor. Besides, the most practical merit of the ZMBR is its rapid startup, stable operation, and tolerance to high influent NLR, which provides a controllable and highly efficient method for the PN of IORW.

### Granular Sludge-Based Cultivation and Its Morphology Structure Change

The granular sludge profited from better sludge settleability than flocculent sludge, which could achieve greater treatment efficiency. Moreover, the relative abundance of ammonium bacteria was directly affected by the nitrogen removal and the living environment of sludge might also be an important factor. Meanwhile, the different microbial structures would bring different excreta that might have positive or negative effects on the surface of sludge. This still needs further verification. Since granular sludge has different spatial distribution in the reactor resulting that a part of granular sludge float on the liquid and another part and floc sludge at the bottom of the reactor. This study disagrees with the opinion of dosing Fe^2+^ can suppress the flotation problem (Wang et al., 2018) because the residual concentration of Fe^2+^ in IORW is still at 15 ± 5 mg/L and believed that the continuous minute bubbles from anammox might be the main reason for the phenomenon of sludge floating. In addition, another study found that using vibration technology is an effective and quick method to form settleable granules (Zhang et al., 2019). The floc particle sludge should be formed from granular collision or decomposition. The efficient anammox consisted of these three different spatial distribution granular sludge and microorganism communities presenting different abundances.

**FIGURE 6:**
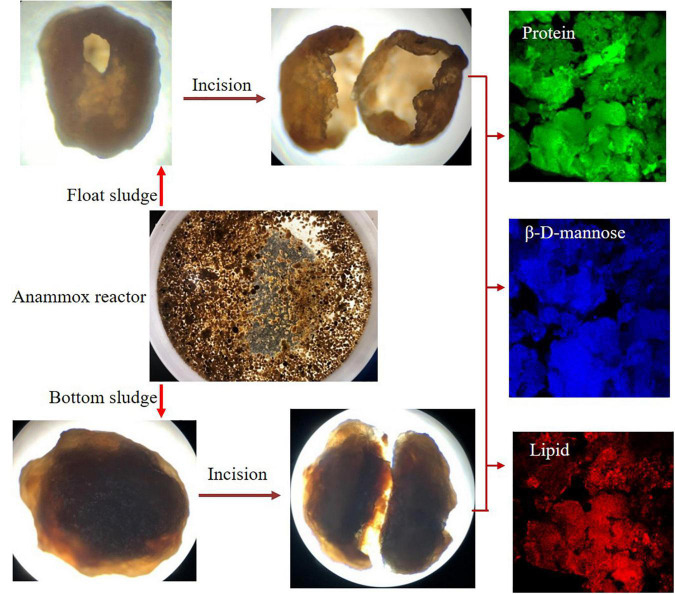
Morphology structure and extracellular polymeric substance (EPS) detection of granular sludges.

Using CLSM observation technology can simultaneously exhibit the distribution and the relative abundance of different EPSs on the granular sludge. As shown in Figure 6, the fluorescence intensity indicated that proteins, lipids, and β-D-mannose were erratically distributed on the granular sludge and the proteins were patchily distributed on the granular sludge (the green color). Weaker fluorescence was found in lipid and β-D-mannose detection compared to the proteins. The EPS distribution revealed that the higher relative abundance of *Ca.* K. stuttgartiensis on granular sludge the better sludge settleability was maintained. Besides, EPSs might play a crucial role during fast anammox bacteria enrichment, which was different from the granular sludge with a low abundance of anammox bacteria (Ni et al., 2015). In addition, the aggregation property from EPS was usually applied to support the anammox bacteria preferably attached to the surface of sludge (Jia et al., 2017). Recent studies reveal that EPSs can enhance the NRR (Liu et al., 2018). EPS might contribute to the anammox bacteria attached to the surface of sludge. As a common carrier, granular sludge provides a suitable environment for anammox bacteria and is beneficial to the nitrogen removal of IORW. According to the microscopic image, a small cavity was common in float sludge in the cultivation phase, while the granular sludge at the bottom of the reactor was a solid sphere. The potential explanation was that ammonium and nitrite were simultaneously removed by anammox bacteria, which would produce a lot of gas on the surface of granular sludge, which can destroy the surface structure. Therefore, floc particles were released from the inside of the granular sludge, which might possible formatted new granular sludge because of the adhesion from abundant EPSs.

## Conclusion

The stable PN was maintained in a ZMBR and obtained 2.78 kg/(m^3^⋅day) in NLR and 1.40 kg/(m^3^⋅day) in NPR by influent IORW with ${\text{NH}}_{4}^{+}$-N concentration at 803 mg/L treatment. Granular sludge was taken from a papermaking wastewater treatment process can be used as a bacterial carrier and *Ca.* K. stuttgartiensis gradually dominant on the sludge. Moreover, the morphology structure and EPSs distribution on granular sludge were investigated resulting that anammox bacteria activities and floc particles released from the inside of the granular sludge being an important reason for sludge floating. Based on the recycle utilization of granular sludge in anammox combined with ZMBR (PN-anammox) can obtain NRR was 2.33 kg/(m^3^⋅day). Because of low-cost and high-efficient, this combined process would have great potential for IORW treatment and larger-scale engineering implementation.

## Data Availability Statement

The original contributions presented in the study are included in the article/supplementary material, further inquiries can be directed to the corresponding author.

## Author Contributions

X-HF, X-JW, and H-HZ: conceptualization and methodology. X-HF, KD, and Z-QZ: project administration and data curation. X-HF and KD: original draft. X-HF and H-YZ: review and editing. X-HF, Y-PL, and H-XL: supervision. All authors have contributed to the article and approved the submitted version of the manuscript.

## Conflict of Interest

The authors declare that the research was conducted in the absence of any commercial or financial relationships that could be construed as a potential conflict of interest.

## Publisher’s Note

All claims expressed in this article are solely those of the authors and do not necessarily represent those of their affiliated organizations, or those of the publisher, the editors and the reviewers. Any product that may be evaluated in this article, or claim that may be made by its manufacturer, is not guaranteed or endorsed by the publisher.
